# Eight Americas: Investigating Mortality Disparities across Races, Counties, and Race-Counties in the United States

**DOI:** 10.1371/journal.pmed.0030260

**Published:** 2006-09-12

**Authors:** Christopher J. L Murray, Sandeep C Kulkarni, Catherine Michaud, Niels Tomijima, Maria T Bulzacchelli, Terrell J Iandiorio, Majid Ezzati

**Affiliations:** 1 Harvard School of Public Health, Boston, Massachusetts, United States of America; 2 Harvard University Initiative for Global Health, Cambridge, Massachusetts, United States of America; 3 Center for Population and Development Studies, Harvard University, Cambridge, Massachusetts, United States of America; 4 University of California San Francisco, San Francisco, California, United States of America; University of California San Francisco, United States of America

## Abstract

**Background:**

The gap between the highest and lowest life expectancies for race-county combinations in the United States is over 35 y. We divided the race-county combinations of the US population into eight distinct groups, referred to as the “eight Americas,” to explore the causes of the disparities that can inform specific public health intervention policies and programs.

**Methods and Findings:**

The eight Americas were defined based on race, location of the county of residence, population density, race-specific county-level per capita income, and cumulative homicide rate. Data sources for population and mortality figures were the Bureau of the Census and the National Center for Health Statistics. We estimated life expectancy, the risk of mortality from specific diseases, health insurance, and health-care utilization for the eight Americas. The life expectancy gap between the 3.4 million high-risk urban black males and the 5.6 million Asian females was 20.7 y in 2001. Within the sexes, the life expectancy gap between the best-off and the worst-off groups was 15.4 y for males (Asians versus high-risk urban blacks) and 12.8 y for females (Asians versus low-income southern rural blacks). Mortality disparities among the eight Americas were largest for young (15–44 y) and middle-aged (45–59 y) adults, especially for men. The disparities were caused primarily by a number of chronic diseases and injuries with well-established risk factors. Between 1982 and 2001, the ordering of life expectancy among the eight Americas and the absolute difference between the advantaged and disadvantaged groups remained largely unchanged. Self-reported health plan coverage was lowest for western Native Americans and low-income southern rural blacks. Crude self-reported health-care utilization, however, was slightly higher for the more disadvantaged populations.

**Conclusions:**

Disparities in mortality across the eight Americas, each consisting of millions or tens of millions of Americans, are enormous by all international standards. The observed disparities in life expectancy cannot be explained by race, income, or basic health-care access and utilization alone. Because policies aimed at reducing fundamental socioeconomic inequalities are currently practically absent in the US, health disparities will have to be at least partly addressed through public health strategies that reduce risk factors for chronic diseases and injuries.

## Abstract 2

US mortality rates were calculated according to "race-county" units and divided into the "eight Americas", across which there are enormous disparities in life expectancy.

## Editors' Summary

**Background.:**

It has been recognized for a long time that the number of years that people in the United States can expect to live (“life expectancy”) varies enormously. For example, white Americans tend to live longer than black Americans, and life expectancy is much greater in some of the roughly 3,000 counties of the US than it is in others. However, there is a lack of information and understanding on how big a part is played in “health inequalities” by specific diseases and injuries, by risk factors (such as tobacco, alcohol, and obesity), and by variations in access to effective health care.

**Why Was This Study Done?:**

The researchers wanted to find a way of dividing the people of the US into groups based on a small number of characteristics—such as location of county of residence, race, and income—that would help demonstrate the most important factors accounting for differences in life expectancy.

**What Did the Researchers Do and Find?:**

The researchers used figures from the US Census Bureau and the National Center for Health Statistics to calculate mortality (death) rates for the years 1982–2001. They took note of the county of residence and of the race of all the people who died during that period of time. This enabled them to calculate the mortality rates for all 8,221 “race-county units” (all of the individuals of a given race in a given county). They experimented with different ways of combining the race-counties into a small and manageable number of groups. They eventually settled on the idea of there being “eight Americas,” defined on the basis of race-county, population density, income, and homicide rate. Each group contains millions or tens of millions of people. For each of the eight groups the researchers estimated life expectancy, the risk of mortality from specific diseases, the proportion of people who had health insurance, and people's routine encounters with health-care services. (The researchers also created maps of life expectancies for the US counties.) They describe their eight Americas as follows: Asians, northland low-income rural whites, Middle America, low-income whites in Appalachia and the Mississippi Valley, western Native Americans, black Middle America, low-income southern rural blacks, and high-risk urban blacks.

Many striking differences in life expectancy were found between the eight groups. For example, in 2001, the life expectancy gap between the 3.4 million high-risk urban black males and the 5.6 million Asian females was nearly 21 years. Within the sexes, the life expectancy gap between the best-off and the worst-off groups was 15.4 years for males (Asians versus high-risk urban blacks) and 12.8 years for females (Asians versus low-income rural blacks in the South). The causes of death that were mainly responsible for these variations were various chronic diseases and injury. The gaps between best-off and worst-off were similar in 2001 to what they were in 1987.

**What Do These Findings Mean?:**

Health inequalities in the US are large and are showing no sign of reducing. Social and economic reforms would certainly help change the situation. At the same time, the public health system should also improve the way in which it deals with risk factors for chronic diseases and injuries so that groups with the highest death rates receive larger benefits.

**Additional Information.:**

Please access these Web sites via the online version of this summary at http://dx.doi.org/10.1371/journal.pmed.0030260.

- A Perspective article by Gregory Pappas in this issue of *PLoS Medicine* (DOI: 10.1371/journal.pmed.0030357) discusses the methods of this piece of research and the findings
- The American Medical Students' Association deals with the question “What are Health Disparities?” on its web site
- The National Institutes of Health's “Strategic Research Plan to Reduce and Ultimately Eliminate Health Disparities” may be seen at the NIH web site
- The Office of Minority Health at the Centers for Disease Control and Prevention has a Web page called “Eliminating Racial and Ethnic Health Disparities”
- The issue of health inequalities in the US has also been dealt with by the Robert Wood Johnson Foundation

## Introduction

Health disparities in the United States have been the subject of extensive critical scrutiny and analysis. Multiple investigations have documented the consistent gap in all measures of mortality by race, particularly between black and white Americans [1–5]. Researchers have also drawn attention to substantial disparities in mortality and functional health status nationally and within race groups in relation to income, social class, education, and community characteristics [6–16]. Inequalities in insurance coverage, health-care access and utilization, and more recently in quality of care have also been investigated [17–22]. The Department of Health and Human Services has launched its Initiative to Eliminate Racial and Ethnic Disparities in Health, with programs focused on a number of diseases including cardiovascular disease, HIV, and diabetes.

**Figure 1:**
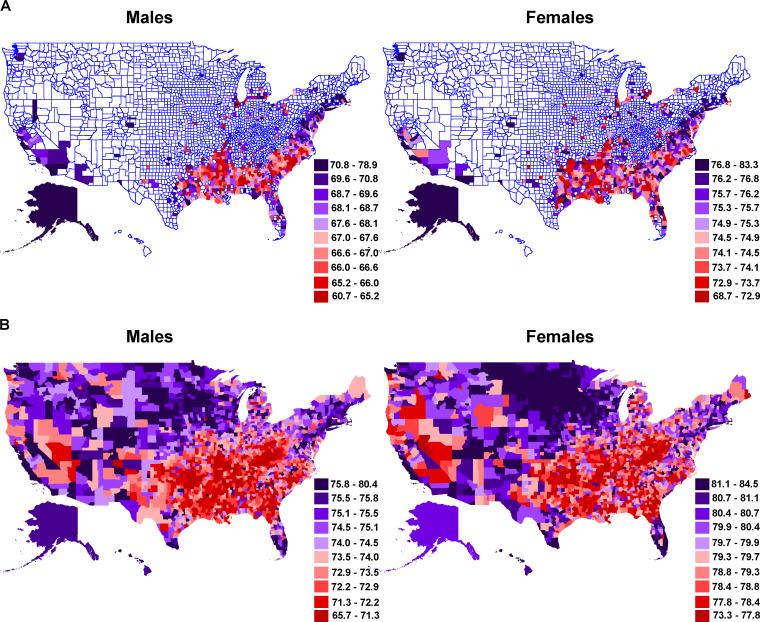
County Life Expectancies by Race Deaths were averaged for 1997–2001 to reduce sensitivity to small numbers and outliers. (A) Life expectancy at birth for black males and females. Only counties with more than five deaths for any 5-y age group (0–85) were mapped, to avoid unstable results. (B) Life expectancy at birth for white males and females.

Life expectancy by race in the US in 2001 ranged from 86.7 for Asian females to 68.7 for black males, a gap of 18 y. Analysis of life expectancy by county of residence and by the combination of race and county of residence (referred to as “race-county” in this paper) demonstrates even larger disparities [23]. County-level analysis of mortality for 1997–2001 (pooled over 5 y to increase sample size) demonstrates a 22.5-y gap in life expectancy between males in southwest South Dakota and females in Stearns County, Minnesota (see Dataset S1 for life expectancy by county). When race-county combinations are considered, life expectancy disparities are dramatically larger. For example, Native American males in the cluster of Bennet, Jackson, Mellette, Shannon, Todd, and Washabaugh Counties in South Dakota had a life expectancy of 58 y in 1997–2001, compared to Asian females in Bergen County, New Jersey, with a life expectancy of 91 y, a gap of 33 y (see also Figure 1). Mortality inequalities in subgroups within race-counties, such as those defined based on socioeconomic status (SES), may be even larger. Because of small sample size and the absence of individual-level linked data needed to study race-county-SES combinations, it is currently not possible to study mortality patterns within race groups in small geographic areas, or even states. The largest measurable gaps observed in the US to-date are those revealed by examining the inequalities across race-county groups.

Formulating effective policies and programs to ameliorate health inequalities requires an understanding of the interrelated causes of mortality disparities, specific interventions to mitigate these causes, and intervention delivery mechanisms [24,25]. Efforts to characterize the contributions of specific diseases and injuries, risk factors such as tobacco, alcohol, or obesity, access to effective health care, and the broader socioeconomic determinants of health and disease are severely hampered by data limitations: the analysis of mortality by age and disease for specific race-counties is affected by the small numbers of deaths, such that even pooling data for 10 y or more does not provide sufficient person-years of observation to draw stable and robust conclusions for some diseases, especially by age. Data on risk factor exposure and health-care access and utilization are even more limited, as there are almost no sources of information on these variables by race and county.

In order to investigate the causes of the observed race-county mortality disparities, within the limitations posed by sample size, we have divided US race-counties into eight subgroups based on a number of sociodemographic and geographical variables, referred to as the “eight Americas”. In addition to examining the role of specific diseases in age-specific and all-age mortality disparities, the eight Americas analysis identifies distinct subgroups, based on a small number of sociodemographic and geographical indicators, towards whom public health and medical interventions may be targeted. We emphasize that the grouping of US race-counties into the eight Americas in our analysis is not the only division that could be used for understanding the large mortality inequalities across racecounties. This grouping however reveals important variations in total as well as age-, sex-, and disease-specific mortality that call for further investigation of causes and potential interventions.

## Methods

### Definition of the Eight Americas

**Figure 2:**
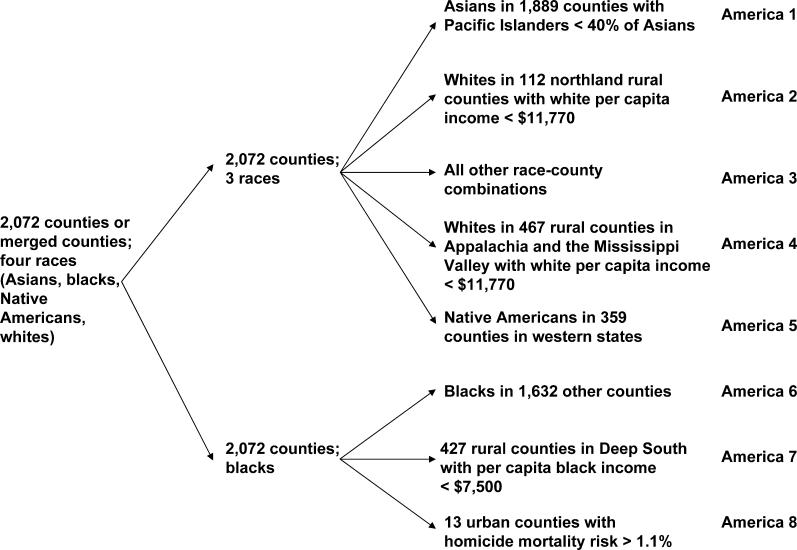
Construction of the Eight Americas from 8,288 Race-County Units

We estimated life expectancy for eight subgroups of the US population, which we refer to as the “eight Americas.” The building blocks for the eight Americas were a combination of race and county of residence. The race-county units were combined based on a number of socioeconomic and geographical indicators, including the location of the county of residence, population density, race-specific county-level per capita income, and cumulative homicide rate (Table 1; Figure 2).

**Table 1:**
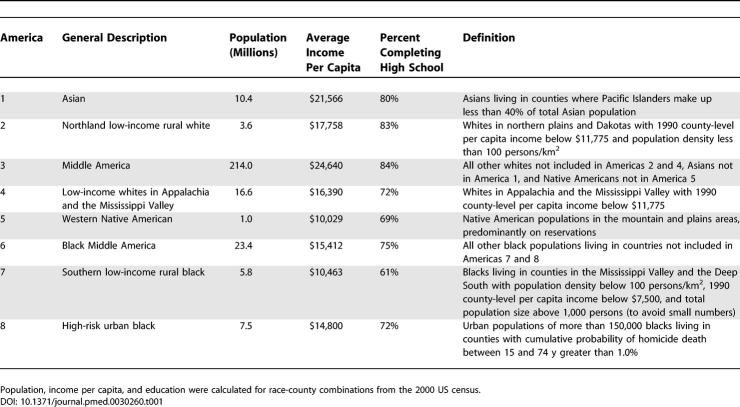
Definitions and Basic Sociodemographic Characteristics of the Eight Americas

We arranged the 3,141 US counties into 2,072 individual or merged county units. There were two reasons for forming merged county units. (1) To avoid very small county populations and numbers of deaths, smaller counties were merged with adjacent counties to form units with total population of at least 10,000 males and 10,000 females. (2) Merged county units were also formed where necessary to account for changes in county status and county lines, such as formations of new counties and incorporation of independent cities (which are officially equivalent to counties) into surrounding counties. The result was a consistent set of 2,072 individual or merged county units that represent the same physical land areas from 1980 through the present. We then divided the entire US population (the race-county units) into eight distinct subgroups, the eight Americas, based on the location of the county of residence, population density, per capita income of the county of residence in 1990, and cumulative homicide rate (averaged between 1991 and 2001 to reduce sensitivity to outlier years) (Table 1; Figure 2). We used a population density of greater than 100 persons/km^2^ to distinguish urban from rural areas. Low-income race-county combinations were defined as those below the national median of race-specific county-level per capita income. Cumulative homicide rate was used as an indicator of deterioration of social institutions and neighborhood cohesion. These factors have been found to adversely affect health outcomes above and beyond individual characteristics [26,27]. High-risk urban areas were defined as those where the cumulative probability of homicide death between the ages of 15 and 74 exceeded the 95th percentile of all US counties, or 1.1%.

### Data Sources for Population, Mortality, and Sociodemographic Indicators 

#### Bureau of the Census population estimates and National Center for Health Statistics bridged-race population estimates.

For 1982–1989, we interpolated age-, sex-, race-, and county-specific population figures using the 1980 and 1990 censual figures, assuming a constant growth rate. For 1990–2001, we used the bridged-race population estimates, released by the National Center for Health Statistics (NCHS), to be consistent with race categories used in mortality registration [28]. Race was defined according to the 1977 US Office of Management and Budget definition of the four race groups (Asians, blacks, Native Americans, and whites), which has been preserved in the bridged and censual population estimates. Race-specific county income was from the 1990 census [29].

#### NCHS mortality statistics.

All deaths occurring in the US are reported to the NCHS, with causes of deaths coded to follow the International Classification of Disease system. NCHS data also include county of residence (matched to the US census), race, sex, and age. Public-use mortality files, available through the NCHS [30] or the National Bureau of Economic Research, do not provide county identifiers for deaths in counties with fewer than 100,000 people. We obtained county identifiers for all deaths for years 1982 through 2001 through a special request to the NCHS. County identifiers for years after 2002 were not provided to us because of changes in NCHS policy. NCHS race categories were collapsed into the four Office of Management and Budget categories. A very small proportion (<0.001%) of all deaths were classified as “other race.” We assumed these deaths to be among Asians, the group with the best mortality experience, in order not to overestimate disparities.

Despite the fact that we used the NCHS bridged-race population estimates, which are estimated for consistency of race definitions with those in death certificates and mortality statistics, there may be differential under- or overestimation of race-specific population and mortality. This can occur because race is recorded by individuals or their families in the census (population) and by the certifying physician or funeral facility on the death certificate (mortality). This differential recording system is a potential source of bias in life expectancy, especially for the smaller race groups: Native Americans and Asians [1,31]. The bias for Native Americans observed in national data is unlikely to affect our estimates because the grouping used in the eight Americas distinguishes those Native Americans who primarily live on or near reservations (America 5), and are hence less likely to be undercounted, from the remaining Native Americans, who are included in America 3. For Asians, this source of bias was addressed by adjusting population and mortality for differential underestimation using the National Mortality Followback Survey [1,31]. Following Hahn and Eberhardt [1], age- and sex-specific correction factors were applied to population and mortality figures for Asians (America 1), with the excess mortality or population coming from Middle America (America 3), the group to which Asians are most likely to be misclassified.

### Data Sources on Health Plan Coverage and Health-Care Utilization

Data on health plan coverage and health-care utilization were from the Behavioral Risk Factor Surveillance System (BRFSS). The BRFSS is an annual cross-sectional telephone survey conducted by the Centers for Disease Control and Prevention and state health departments. The BRFSS uses a multistage cluster design based on random-digit dialing to select a representative sample from each state's noninstitutionalized civilian residents aged 18 y and older, and is described in detail elsewhere [32,33] and in online documentation (http://www.cdc.gov/brfss). The BRFSS questionnaire primarily focuses on personal risk behaviors and exposures, but also includes questions on health plan coverage and utilization of care. The BRFSS data for 2001–2003 were averaged to reduce sensitivity to interannual fluctuations. Counties were matched to the appropriate America using the standardized Federal Information Processing Standards (FIPS) county codes. Counties with unidentifiable codes (<1% of all counties) were excluded from the analysis. The BRFSS survey instrument on health-care access asks about health-care coverage status including health insurance, prepaid plans such as HMOs, and government programs such as Medicare or Medicaid. The BRFSS also asks about time since the last routine checkup. BRFSS data from the latest 2–3 y before the analysis were used: 2001–2003 for health-care coverage and 2001–2002 for checkup (this variable was not included in the 2003 BRFSS).

### Data for International Comparisons

Data on life expectancy and probabilities of death (all-cause and disease-specific) for international comparisons were from the Global Burden of Disease databases, maintained by the World Health Organization [34,35].

### Analysis Methods

Period all-cause and cause-eliminated life expectancies and probabilities of death were calculated using standard demographic life-table techniques [36].

## Results

### Definitions of the Eight Americas

Table 1 summarizes the characteristics of the eight Americas (see also Figures S1 and S2). America 1 consists of Asians, excluding those living in counties where Pacific Islanders make up more than 40% of the total Asian population. Asians in the latter group of counties were included in America 3. The 10.1 million Asians in America 1 have levels income and education almost exactly even with the national average. America 2 consists of northland (Minnesota, the Dakotas, Iowa, Montana, and Nebraska) low-income rural white populations, with income and education below the national average. America 3 is Middle America, the large fraction of the US population that has slightly above-average per capita income and education. This group largely consists of white Americans (98% of America 3), but also includes small numbers of Asians and Native Americans living in counties that are not included in Americas 1 and 5. America 4 consists of low-income white populations in Appalachia and the Mississippi Valley, 30% of whom have not completed high school. America 5 is made up of Native Americans living in the West, excluding the West Coast. The majority of this group lives on or near reservations, mostly in the Four Corners region (where the borders of Arizona, Colorado, New Mexico, and Utah meet) or the Dakotas. America 7 comprises low-income rural blacks in the Mississippi Valley and the Deep South. America 8 includes blacks living in high-risk urban environments. America 6 represents the rest of black America, living mostly in urban or semi-urban counties.

### Mortality Experiences of the Eight Americas

**Figure 3:**
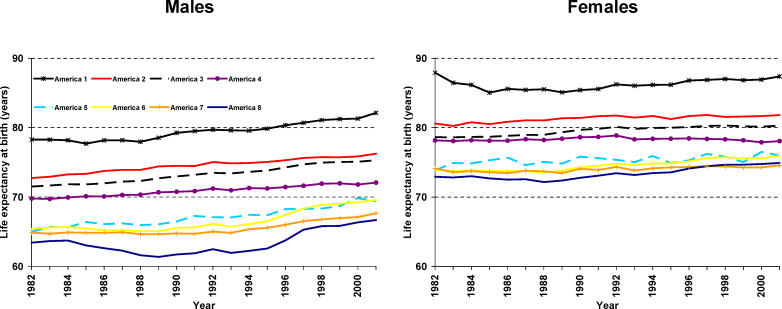
Life Expectancy at Birth in the Eight Americas (1982–2001) Estimates for Americas 1 and 3 have been adjusted for differential underestimation of population and mortality among Asians (see Methods).

**Figure 4:**
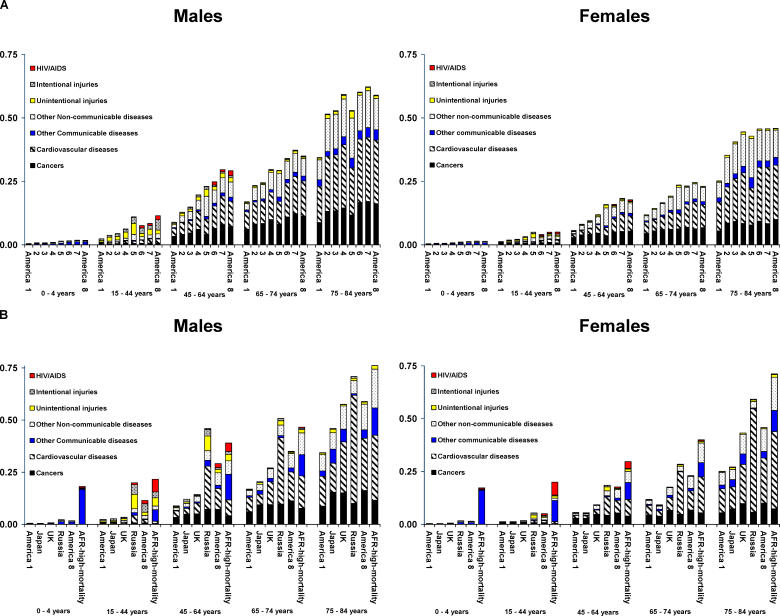
Probability of Dying in Specific Age Ranges in the Eight Americas (A) Probability of death by sex, age, and disease for the eight Americas in 2001. (B) Probability of death by sex, age, and disease for Americas 1 and 8 compared to Japan, UK, the Russian Federation, and high-mortality countries in sub-Saharan Africa (AFR-high-mortality; made up largely of countries in West Africa and excluding countries with very high mortality due to HIV/AIDS) in 2001. Results are not shown for ages 5–14 y because there are few deaths in this age range in the US.

Figure 3 summarizes the mortality experiences for the eight Americas between 1982 and 2001, divided into broad age groups and diseases in Figure 4A for 2001. Asian Americans have sustained extraordinary advantage over the nearest groups, the northland low-income white rural populations (America 2; 5.9 y higher life expectancy for males and 5.6 y for females in 2001; 5.5 and 7.3 y, respectively, in 1982) and Middle America (America 3; 6.8 y higher life expectancy for males and 7.1 y for females in 2001; 6.8 and 9.3 y, respectively, in 1982). Therefore, although many second-generation (US-born) Asians have entered the adult cohorts, the sustained gap between America 1 and other groups has not narrowed over the last 20 y (see also Singh and Miller [4]).

The gap between the life expectancy of the 3.4 million black males in high-risk urban areas in America 8 and the life expectancy for the 5.5 million Asian females in America 1 in 2001 was 20.7 y. Within the sexes, the gap between the best-off and the worst-off groups was 15.4 y for males (Asians versus blacks in high-risk urban areas) and 12.8 y for females (Asians versus low-income southern rural blacks). These gaps are 2.4 and 2.8 times those between white and black life expectancies for the nation as a whole for males and females, respectively. During the late 1980s and early 1990s, the gap between Americas 1 and 8 for males widened significantly, mostly because of higher HIV and homicide death rates. Excluding this period, the gap in male life expectancy between Americas 1 and 8 increased by half a year between 1982 and 2001. Excluding the first few years of the 1980s, when there was an unexplained decline in life expectancy of Asian females, the gap in female life expectancy between Americas 1 and 8 declined by 2.5 y; the gap in life expectancy between Asian females and low-income southern rural black females (America 7) decreased by 1 y (from 13.8 to 12.8 y).

The 12.8-y gap in life expectancy between females in Americas 1 and 7 is approximately the same as the gap between Japan, with the highest national life expectancy for females in 2001 (84.7 y), and Fiji, Nicaragua, and Lebanon [34]. Asian females in the US have a life expectancy that is 3 y higher than that of females in Japan [34]. For males, the 15.4-y gap in life expectancy between Asians (America 1) and high-risk urban blacks (America 8) is the same as between Iceland, with the highest national male life expectancy in 2001 (78.2 y), and Sao Tome, Belarus, and Uzbekistan [34]. In other words, millions of Americans, distinctly identified by their sociodemographic characteristics and place of residence, have life expectancies that are similar to some low-income developing countries (see also Figure 4B).

The eight Americas classification reveals that within the white population there is a wide variation in health experience that cannot be explained by differences in average income: low-income white rural populations in Minnesota, the Dakotas, Iowa, Montana, and Nebraska (America 2), with a life expectancy of 76.2 and 81.8 y for males and females, respectively, have a substantial advantage over the rest of white America, despite a large income disadvantage. Low-income whites in Appalachia and the Mississippi Valley (America 4), with an average income level similar to that of America 2, have a life expectancy equal to those of Mexico and Panama. The life expectancy gap between whites in America 2 and America 4 was 4.2 and 3.8 y in 2001 for males and females, respectively, comparable to the 6.4- and 4.6-y gaps between whites and blacks as a whole. The gap between whites in America 2 and America 4 has in fact increased since 1982, when it was 3.0 and 2.4 y for males and females respectively; between 1982 and 2001 life expectancy among females in America 4 declined from 78.2 y to 78.1 y.

Because America 3 is the largest subgroup of the US population, we investigated the potential role of sociodemographic and geographical predictors used to define the eight Americas in morality patterns within America 3. County-level analysis of mortality for 1997–2001 shows that average life expectancy for whites in America 3 (98% of America 3 are whites) was 77.1 y, with a standard deviation of 1.7 y. The correlation coefficient between county-level life expectancy and per capita income in 2000 was 0.35. Life expectancy ranged from 76.7 to 78.3 y in the quartiles of county-level per capita income; it ranged from 77.0 to 78.6 y in quartiles of county-level education (based on proportion who have completed high school). The highest county-level education quartile consistently had the highest life expectancy over time, but there was no consistent education gradient for the other three quartiles. There was also no obvious geographical pattern in mortality in America 3 (Figure 1). For example, life expectancy for counties east and west of the Mississippi River was 76.6 and 77.2 y, respectively.

Although at the national level Native Americans seemingly had a life expectancy equal to or higher than whites, Native Americans living in the West, mostly on or near reservations (America 5) and with the lowest per capita income of all the eight Americas had a major mortality disadvantage. Life expectancies for America 5 were 5.9 and 4.3 y lower than Middle America (America 3) for males and females, respectively, in 2001. Cause-of-death analysis for America 5 demonstrates that this Native American population has very high rates of mortality from alcohol-related causes such as road traffic accidents and cirrhosis of the liver, as well as diabetes (Figure 4A). Across the three black Americas (black Middle America, southern low-income rural, and high-risk urban), the gap in life expectancy in 2001 was 1 y for females and nearly 3 y for males. The male gap was as wide as 4 y in the mid-1990s, with the subsequent decline mostly due to reduction in HIV/AIDS mortality.

### Age and Disease Patterns of Mortality in the Eight Americas

Figure 4 shows mortality risk in four age groups (0–4, 15–44, 45–64, and 65–74 y) for the eight Americas, and further compares Americas 1 and 8 with countries and regions at a range of mortality levels including Japan, the United Kingdom, the Russian Federation, and the high-mortality countries of sub-Saharan Africa. Although an important gradient in child mortality remains between America 1 and America 8, a disadvantaged child in the US has an order of magnitude lower risk of death compared to low-income nations in sub-Saharan Africa. Above 70 y of age, disparities in mortality in the eight Americas are also reduced, especially in international comparisons. This pattern is partly because of the generally higher mortality in this age group and possibly also because of cohort effects.

**Figure 5:**
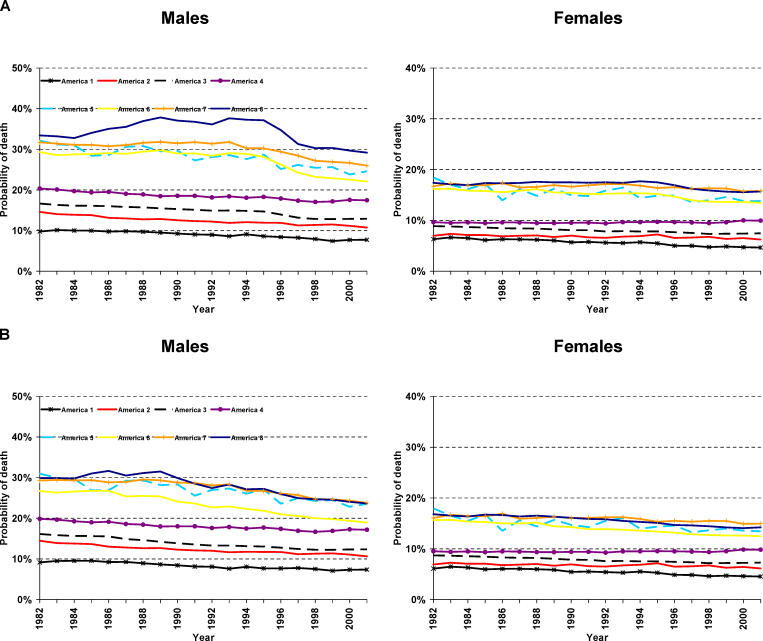
Probability of Death between the Ages of 15 and 59 y in the Eight Americas (A) Probability of death between the ages of 15 and 59 y in the eight Americas from all causes. (B) Probability of death between the ages of 15 and 59 y in the eight Americas after deleting deaths from homicide and HIV.

The mortality disparities in the eight Americas are largest for young (ages 15–44) and middle-aged adults (ages 45–64). In these age groups, blacks living in high-risk urban areas (America 8) have mortality risks more similar to ones in the Russian Federation and sub-Saharan Africa, which are substantially higher than those in America 1, Japan, or the United Kingdom. In 2001, 15-y-old black men and women in high-risk urban areas (America 8) were, respectively, 3.8 and 3.4 times as likely as those in America 1 to die before the age of 60 (Figure 5A), and 4.7 and 3.8 times more likely before the age of 45. The disparity in young and middle-aged adult mortality between America 8 and America 1 has increased since 1982, when the mortality risk ratio was 3.4 and 2.8 for men and women, respectively. The excess young and middle-aged mortality persists after removing the effects of HIV and homicide (Figure 5B). Rather, the major mortality gradients in these age groups are observed for injuries, cardiovascular diseases, and other noncommunicable causes such as liver cirrhosis and diabetes (Figure 4A). Injuries are especially important for the observed mortality gradients between the ages of 15 and 44.

### Health-Care Access and Utilization

**Figure 6:**
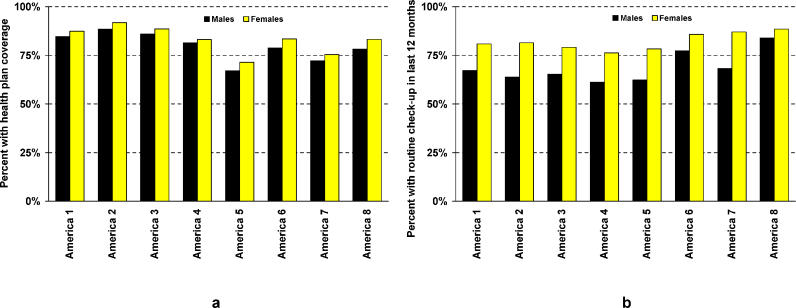
Health Plan Coverage and Health Service Utilization in the Eight Americas

Figure 6A shows self-reported health plan coverage in the eight Americas. Native Americans in the West (America 5) reported the lowest coverage, followed by low-income rural southern blacks (America 7). The highest coverage was in northland white low-income rural populations (America 2), followed by Middle America and Asians (Americas 3 and 1). Basic health system encounter, measured as the fraction reporting a routine checkup in the past 12 mo, also shows relatively small variation across the eight Americas. For both males and females, the rates of routine checkup are slightly higher in Americas 6, 7, and 8, the groups with mortality disadvantage.

## Discussion

The eight Americas in this paper consist of distinct subgroups of the US population defined based on a small number of sociodemographic and geographical indicators including race, the location of the county of residence, population density, race-specific county-level per capita income, and cumulative homicide rate. Put in a global context, the disparities in mortality experiences among the eight Americas, each consisting of millions or tens of millions of Americans, are enormous. The eight Americas analysis indicates that ten million Americans with the best health have achieved one of the highest levels of life expectancy on record, 3 y better than Japan for females and 4 y better than Iceland for males. At the same time, tens of millions of Americans are experiencing levels of health that are more typical of middle-income or low-income developing countries. These poor levels of health occur in areas throughout the country. The health disparities among the eight Americas cannot be explained by single causes of death such as homicide or HIV. Nor are the largest sources of disparity in children and the elderly. The mortality disparities are most concentrated in young and middle-aged males and females, and are a result of a number of chronic diseases and injuries with well-established risk factors.

Trends in life expectancy show that neither the relative ordering nor the absolute levels of life expectancy disparities among the eight Americas decreased between 1982 and 2001. During the late 1980s and early 1990s, the life expectancy gap between America 8 and the remaining groups widened significantly for males, mostly because of HIV and homicide rates. In this overall picture of stable disparities, there have been specific groups whose mortality has worsened. For example, the life expectancy of low-income white females in Appalachia and the Mississippi Valley declined between 1982 and 2001.

The most important limitation of the data used for our analysis is that reported race in the census, used for population estimates, may be different from race in mortality statistics, where race may be reported by the family, the certifying physician, or the funeral director [1,31]. Sensitivity to differential race reporting would be largest for those groups with small population and a relatively large proportion of mixed-race individuals, mainly the Native Americans and Asians [1,31]. In these groups, it is more likely for race to be reported as Native American or Asian in the census than it is in the death certificate; hence, the differential underestimation of deaths and population results in bias in the form of a net underestimation of mortality [1,31]. The mortality and population for Asians were corrected for differential underestimation, using the National Mortality Followback Survey [1,31] (Correction using the National Longitudinal Mortality Study would have given virtually identical estimates: 12% underreporting of mortality in the National Mortality Followback Survey versus 13% in the National Longitudinal Mortality Study [31]). For Native Americans, misreporting race on death certificates is most important where mixed races exist and Native Americans form a small proportion of the population (e.g., in California). The grouping used in the eight Americas distinguished between those Native Americans who primarily live on or near reservations (America 5) and the remaining Native Americans (included in America 3), and is therefore robust to this error because living on or near reservations increases the likelihood of correct race recording on death certificates. Any residual bias would be in the form of net underestimation of mortality as described above and as observed nationally for Native Americans [31]. Mortality underestimation would in turn imply that life expectancy is even *lower* in America 5, and hence further magnify the findings of the above analysis on mortality disadvantage in America 5.

A secondary source of bias for Asians may be back-migration of first-generation immigrants, who return to their home countries due to illness. This would lead to an underreporting of deaths for Asians, and overestimation of life expectancy in America 1. To examine the effect of this source of bias, we repeated the analysis restricting the sample to US-born Asians. Excluding the two states (Alaska and Hawaii) whose Asian population is entirely in America 3 (and not in America 1), US-born Asian females and males had life expectancies at birth that were, respectively, 5.1 and 1.6 y higher than those for combined US-born and foreign-born Asians together, consistent with previous findings on immigrant populations [4]. Lower mortality in US-born Asians confirms that the patterns in Figures 3–5 are not a result of unrecorded mortality due to return migration among Asian populations; rather, they represent a real mortality advantage in America 1.

The findings on persistent health disparities in the eight Americas raise the question of why, as a society, we have failed to narrow health gaps between distinct and large subgroups of the US population. Opportunities and interventions to reduce health inequalities include (1) reducing socioeconomic inequalities, which are the distal causes of health inequalities, (2) increasing financial access to health care by decreasing the number of Americans without health plan coverage, (3) removing physical, behavioral, and cultural barriers to health care, (4) reducing disparities in the quality of care, (5) designing public health strategies and interventions to reduce health risks at the level of communities (e.g., changes in urban/neighborhood design to facilitate physical activity and reduce obesity), and (6) designing public health strategies to reduce health risks that target individuals or population subgroups that are not necessarily in the same community (e.g., tobacco taxation or pharmacological interventions for blood pressure and cholesterol).

Important research in the past few decades has illustrated the critical role of individual and community-level socioeconomic factors, be it in absolute or relative terms, in health outcomes [11,24,37,38]. In addition to (or in the absence of, as is currently the case in the US) systematic policies for reducing socioeconomic inequalities, public health and health care provide instruments for addressing inequalities in health outcomes. Much of the health policy agenda in the US is currently focused on health insurance coverage for the nearly 44 million Americans (15% of the population) who lack health insurance [39,40]. Although increasing insurance coverage and access to care would most likely contribute to narrowing disparities across the eight Americas, the available data (Figure 6) suggest that the variation in health plan coverage across the eight Americas is small relative to the very large gradient in health outcomes. It is likely that expanding insurance coverage alone would still leave huge disparities in young and middle-aged adults. A shortcoming of the BRFSS data on health insurance and health-care utilization, however, is that they do not provide any insight into the likely contribution of variation in quality of care to the disparities across the eight Americas.

**Figure 7:**
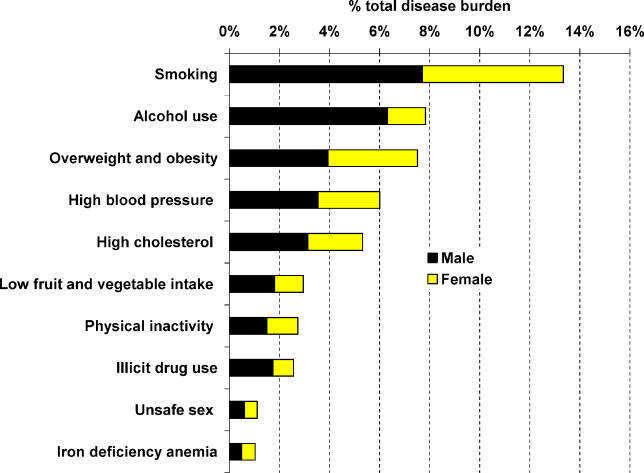
Burden of Disease Attributable to the Ten Leading Risk Factors in the very-low-mortality countries of the Region of Americas The estimates refer to the Global Burden of Disease epidemiological region that includes Canada, Cuba, and the US [45]; more than 85% of this region's population live in the US and most data sources apply to the US.

The diseases with the largest contribution to mortality disparities across the eight Americas are chronic diseases and injuries with well-established risk factors, including alcohol use, tobacco smoking, overweight and obesity, and elevated blood pressure, cholesterol, and glucose. These risk factors are also the leading cause of burden of disease in the nation as a whole (Figure 7). An important question, therefore, is the distributions of exposure to these risks in the eight Americas, and the fraction of disease-specific and all-cause mortality attributable to their hazardous effects. This is particularly relevant for combinations of risk factors that together account for large proportions of many chronic diseases [41,42]. Definite estimates of the contributions of risk factors to health inequalities require analyses for race-county combinations, which are not readily possible using currently available data: among data sources on risk factors, the BRFSS allows subnational analysis but relies on self-reported exposure. Although self-reported exposure is the common metric for risks such as tobacco smoking and alcohol use, it is subject to bias for overweight and obesity and for elevated blood pressure, cholesterol, and glucose because of individual reporting behavior and because individuals may not be aware of their blood pressure, cholesterol, and glucose status. The National Health and Nutrition Examination Survey provides measurements of this latter group of risks, but does not include sufficient geographical detail for analysis at the county or even state level [25]. Methods for combining the two data sources to obtain estimates for population subgroups based on race and place of residence are required [43]. If analysis of risk factors illustrates that a substantial proportion of disparities among the eight Americas are attributable to risk factor exposure, risk factor interventions should be investigated as tools for reducing health inequalities, in the same way that they have been used for achieving aggregate national health benefits. The interventions will likely include both population-wide measures (e.g., tobacco taxation, drinking-and-driving countermeasures, and interventions to reduce public and domestic alcohol-induced violence) and personal interventions (e.g., pharmacological interventions for blood pressure and cholesterol).

The traditional emphasis of the US health system has been on children and the elderly, as, for example, illustrated by the low levels of resources devoted to injury prevention and tobacco control compared with immunization [44]. This emphasis may have partly contributed to substantially lower disparities in these age groups relative to young and middle-aged adults. On the other hand, the emphasis on children and the elderly has treated many of the diseases that are important contributors to young and middle-aged adult health disparities, and their risk factors, as either the responsibilities of individuals (alcohol, tobacco, obesity, and dietary determinants of blood pressure and cholesterol, like salt) or in the domain of clinical care (blood pressure and cholesterol). A number of important steps are needed to broaden the current perspective. First, there is a need to use systematic epidemiological and economic analyses to identify effective and cost-effective health interventions—whether targeting populations or individuals—that would make the biggest difference to those with the worst health. Given the distinct epidemiological, geographical, and sociodemographic profiles of the eight Americas, the leading interventions may be different for each, although some common core strategies may exist (e.g., common core cardiovascular disease prevention strategies). Second, monitoring systems should be developed by the states and territories to provide local but benchmarked information on the fraction of the population in each community who would benefit from these interventions and are receiving them. Third, information on the delivery of these interventions for different communities should be publicly reported. It is when the public, community and professional groups, media, and politicians focus attention on what is being achieved, and why efforts are working in some places and not others, that the culture of accountability for health outcomes will be strengthened.

## Supporting Information

Dataset S1 Life Expectancy at Birth by County Found at 10.1371/journal.pmed.0030260.sd001 (4 MB XLS).

Figure S1 Geographic Locations for Americas 2, 3, and 4 Found at 10.1371/journal.pmed.0030260.sg001 (549 KB PDF).

Figure S2 Geographic Locations for Americas 6, 7, and 8 Found at 10.1371/journal.pmed.0030260.sg002 (268 KB PDF).

## Abbreviations

BRFSS: Behavioral Risk Factor Surveillance System
NCHS: National Center for Health Statistics
SES: socioeconomic status

## References

[pmed-0030260-b001] Hahn RA, Eberhardt S (1995). Life expectancy in four U.S. racial/ethnic populations: 1990. Epidemiology.

[pmed-0030260-b002] Wong MD, Shapiro MF, Boscardin WJ, Ettner SL (2002). Contribution of major diseases to disparities in mortality. N Engl J Med.

[pmed-0030260-b003] Singh GK, Yu SM (1996). Trends and differentials in adolescent and young adult mortality in the United States, 1950 through 1993. Am J Public Health.

[pmed-0030260-b004] Singh GK, Miller BA (2004). Health, life expectancy, and mortality patterns among immigrant populations in the United States. Can J Public Health.

[pmed-0030260-b005] Cooper R, Cutler J, Desvigne-Nickens P, Fortmann S, Friedman L (2000). Trends and disparities in coronary heart disease, stroke, and other cardiovascular diseases in the United States: Findings of the national conference on cardiovascular disease prevention. Circulation.

[pmed-0030260-b006] Navarro V (1990). Race or class versus race and class: Mortality differentials in the United States. Lancet.

[pmed-0030260-b007] Geronimus AT, Bound J, Waidmann TA, Hillemeier MM, Burns PB (1996). Excess mortality among blacks and whites in the United States. N Engl J Med.

[pmed-0030260-b008] Geronimus AT, Bound J, Waidmann TA (1999). Poverty, time, and place: Variation in excess mortality across selected US populations, 1980–1990. J Epidemiol Community Health.

[pmed-0030260-b009] Hahn RA, Eaker ED, Barker ND, Teutsch SM, Sosniak WA (1996). Poverty and death in the United States. Int J Health Serv.

[pmed-0030260-b010] Isaacs SL, Schroeder SA (2004). Class—The ignored determinant of the nation's health. N Engl J Med.

[pmed-0030260-b011] Adler N, Marmot M, McEwan B, Stewart J (1999). Socioeconomic status and health in industrial nations: Social, psychological, and biological pathways.

[pmed-0030260-b012] Singh GK (2003). Area deprivation and widening inequalities in US mortality, 1969–1998. Am J Public Health.

[pmed-0030260-b013] Muntaner C, Eaton WW, Miech R, O'Campo P (2004). Socioeconomic position and major mental disorders. Epidemiol Rev.

[pmed-0030260-b014] Pappas G, Queen S, Hadden W, Fisher G (1993). The increasing disparity in mortality between socioeconomic groups in the United States, 1960 and 1986. N Engl J Med.

[pmed-0030260-b015] Davey Smith G, Neaton JD, Wentworth D, Stamler R, Stamler J (1998). Mortality differences between black and white men in the USA: Contribution of income and other risk factors among men screened for the MRFIT. MRFIT Research Group. Multiple Risk Factor Intervention Trial. Lancet.

[pmed-0030260-b016] Krieger N, Chen JT, Waterman PD, Rehkopf DH, Subramanian SV (2005). Painting a truer picture of US socioeconomic and racial/ethnic health inequalities: The Public Health Disparities Geocoding Project. Am J Public Health.

[pmed-0030260-b017] Smedley BD, Stith AY, Nelson AR (2003). Unequal treatment: Confronting racial and ethnic disparities in health care.

[pmed-0030260-b018] Bloche MG (2004). Health care disparities—Science, politics, and race. N Engl J Med.

[pmed-0030260-b019] Gornick ME, Eggers PW, Riley GF (2001). Understanding disparities in the use of Medicare services. Yale J Health Policy Law Ethics.

[pmed-0030260-b020] Gornick ME, Eggers PW, Reilly TW, Mentnech RM, Fitterman LK (1996). Effects of race and income on mortality and use of services among Medicare beneficiaries. N Engl J Med.

[pmed-0030260-b021] Epstein AM, Ayanian JZ (2001). Racial disparities in medical care. N Engl J Med.

[pmed-0030260-b022] Goldman DP, Smith JP (2002). Can patient self-management help explain the SES health gradient?. Proc Natl Acad Sci U S A.

[pmed-0030260-b023] Murray CJL, Michaud C, Mckenna MT, Marks JS (1998). US patterns of mortality by county and race: 1965–1994.

[pmed-0030260-b024] Mackenbach JP, Bakker MJ (2003). Tackling socioeconomic inequalities in health: Analysis of European experiences. Lancet.

[pmed-0030260-b025] Otten MW, Teutsch SM, Williamson DF, Marks JS (1990). The effect of known risk factors on the excess mortality of black adults in the United States. JAMA.

[pmed-0030260-b026] Stafford M, Bartley M, Mitchell R, Marmot M (2001). Characteristics of individuals and characteristics of areas: Investigating their influence on health in the Whitehall II study. Health Place.

[pmed-0030260-b027] Stafford M, Marmot M (2003). Neighbourhood deprivation and health: Does it affect us all equally?. Int J Epidemiol.

[pmed-0030260-b028] Ingram DD, Parker JD, Schenker N, Weed JA, Hamilton B (2003). United States Census 2000 population with bridged race categories. Vital Health Stat.

[pmed-0030260-b029] US Census Bureau. 1990 Census Summary Tape File 3.

[pmed-0030260-b030] National Center for Health Statistics (2006). Mortality data, multiple cause-of-death public-use data files.

[pmed-0030260-b031] Rosenberg H, Maurer J, Sorlie P, Johnson N, MacDorman MF (1999). Quality of death rates by race and Hispanic origin: A summary of current research, 1999. Vital Health Stat.

[pmed-0030260-b032] Nelson DE, Holtzman D, Waller M, Leutzinger CL, Condon K (1998). Objectives and design of the Behavioral Risk Factor Surveillance System. Proceedings of the Survey Research Methods Section, American Statistical Association National Meeting (1998).

[pmed-0030260-b033] Remington PL, Smith MY, Williamson DF, Anda RF, Gentry EM (1988). Design, characteristics, and usefulness of state-based behavioral risk factor surveillance: 1981–87. Public Health Rep.

[pmed-0030260-b034] World Health Organization (2002). The world health report 2002: Reducing risks, promoting healthy life.

[pmed-0030260-b035] Mathers CD, Lopez AD, Murray CJL, Lopez AD, Mathers CD, Ezzati M, Jamison DT, Murray CJL (2006). The burden of disease and mortality by condition: Data, methods, and results for 2001. Global burden of disease and risk factors.

[pmed-0030260-b036] Preston SH, Heuveline P, Guillot M (2001). Demography: Measuring and modeling population processes.

[pmed-0030260-b037] Marmot MG (2003). Understanding social inequalities in health. Perspect Biol Med.

[pmed-0030260-b038] Marmot M (2002). The influence of income on health: Views of an epidemiologist. Does money really matter? Or is it a marker for something else?. Health Aff (Millwood).

[pmed-0030260-b039] US Census Bureau (2003). Health insurance coverage in the United States: 2002.

[pmed-0030260-b040] McCanne DR (2004). A national health insurance program for the United States. PLoS Medicine.

[pmed-0030260-b041] Ezzati M, Vander Hoorn S, Rodgers A, Lopez AD, Mathers CD (2003). Estimates of global and regional potential health gains from reducing multiple major risk factors. Lancet.

[pmed-0030260-b042] Yusuf S, Hawken S, Ounpuu S, Dans T, Avezum A (2004). Effect of potentially modifiable risk factors associated with myocardial infarction in 52 countries (the INTERHEART study): Case-control study. Lancet.

[pmed-0030260-b043] Ezzati M, Martin H, Skjold S, Vander HS, Murray CJL (2006). Trends in national and state-level obesity in the USA after correction for self-report bias: Analysis of health surveys. J R Soc Med.

[pmed-0030260-b044] Centers for Disease Control and Prevention (2005). FY 2005 CDC appropriation—Budget by functional area.

[pmed-0030260-b045] Ezzati M, Lopez AD, Rodgers A, Vander Hoorn S, Murray CJL (2002). Selected major risk factors and global and regional burden of disease. Lancet.

