# Correction: Eight Americas: Investigating Mortality Disparities across Races, Counties, and Race-Counties in the United States

**DOI:** 10.1371/journal.pmed.0030545

**Published:** 2006-12-26

**Authors:** Christopher J. L Murray, Sandeep C Kulkarni, Catherine Michaud, Niels Tomijima, Maria T Bulzacchelli, Terrell J Iandiorio, Majid Ezzati

In *PLoS Medicine*, volume 3, issue 9: doi:10.1371/journal.pmed.0030260

## Main Paper

In Table 1, row “America 6,” last column: “countries” should be “counties.”

In Methods, “High-risk urban areas were defined as those where the cumulative probability of homicide death between the ages of 15 and 74 exceeded the 95th percentile of all US counties, or 1.1%.” should be “High-risk urban areas were defined as those where the cumulative probability of homicide death between the ages of 15 and 74 exceeded the 95th percentile of all US counties, or 1.0%.”

In Figure 2, “homicide mortality risk > 1.1%” should be “homicide mortality risk > 1.0%.”

In Results, “10.1 million Asians in America 1” should be “10.4 million Asians in America 1.”

The term “Eight Americas,” an earlier revision of the description of the Eight Americas, and an earlier revision of life expectancies for 2001 were previously published in a commentary in the *American Journal of Preventive Medicine* [1]. This commentary cited the current paper as the technical source of this information, also declared at the time of submission of the current manuscript. This earlier publication should have been cited in the current article, under Introduction, after the sentence “…we have divided US race-counties into eight subgroups based on a number of sociodemographic and geographical variables, referred to as the ‘eight Americas’.”

## Supplementary Data

In Dataset S1 of the Supporting Information, “Washabaugh” county, which appears twice in rows 2439 and 2440, should be dropped in both instances (this county no longer has its own FIPS code as it was incorporated into another county).
