# Supplementary material for: Eight Americas: Investigating Mortality Disparities across Races, Counties, and Race-Counties in the United States
Source: PLoS Med. 2006 Sep 12;3(9):e260. doi: 10.1371/journal.pmed.0030260 (PMC1564165; doi:10.1371/journal.pmed.0030260)

Supplementary Online Material

Figure S-1: Geographic locations for Americas 2, 3, and 4.

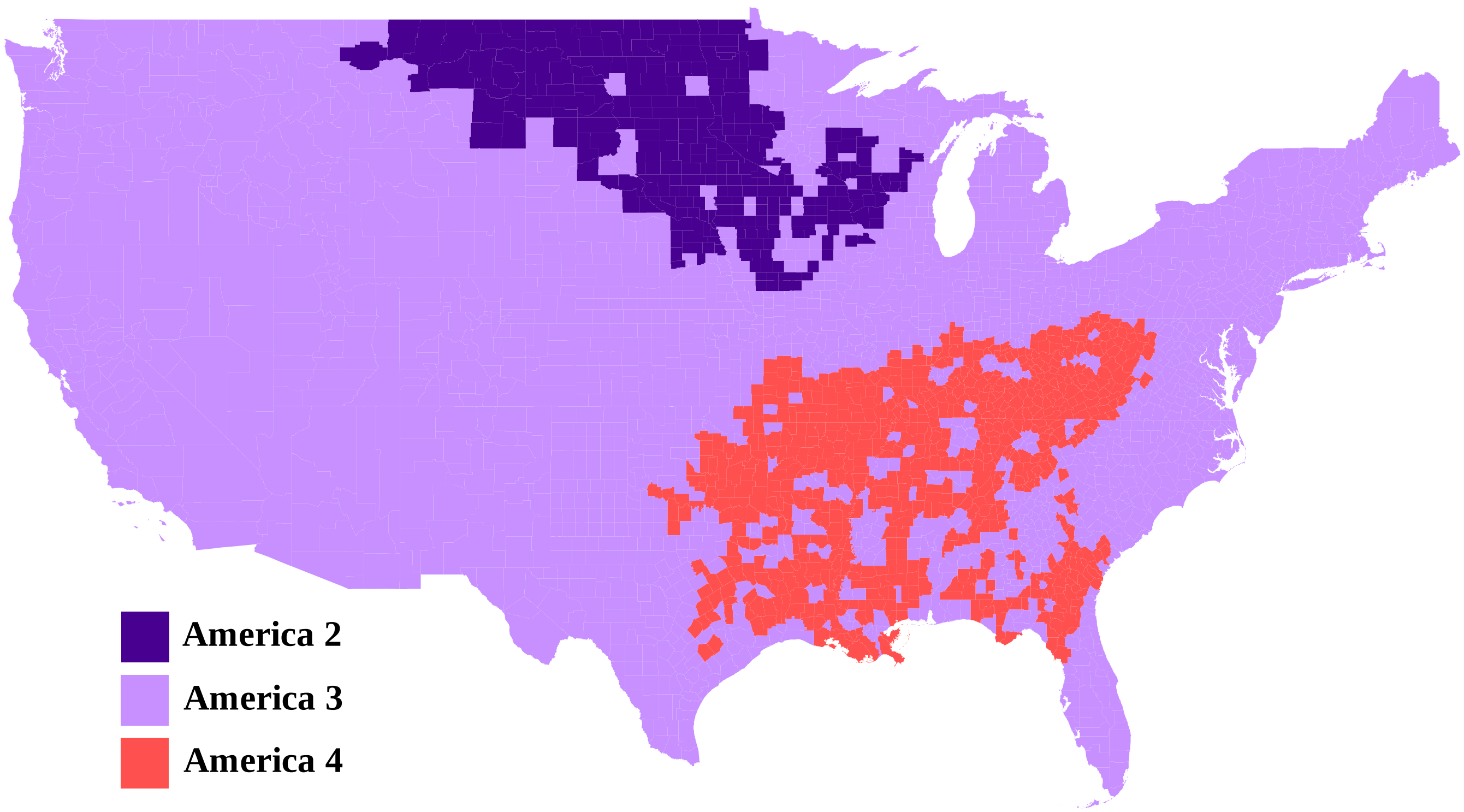

Supplement: Supplementary file 1 [file pmed.0030260.sg001.pdf]
